# Understanding Ciprofloxacin Failure in *Pseudomonas aeruginosa* Biofilm: Persister Cells Survive Matrix Disruption

**DOI:** 10.3389/fmicb.2019.02603

**Published:** 2019-11-13

**Authors:** Anaïs Soares, Valérie Roussel, Martine Pestel-Caron, Magalie Barreau, François Caron, Emeline Bouffartigues, Sylvie Chevalier, Manuel Etienne

**Affiliations:** ^1^GRAM 2.0, EA 2656, Normandie University, UNIROUEN, Rouen, France; ^2^Microbiology Department, Rouen University Hospital, Rouen, France; ^3^EA 4312, LMSM, Normandie University, UNIROUEN, Evreux, France; ^4^Infectious Diseases Department, Rouen University Hospital, Rouen, France

**Keywords:** *Pseudomonas aeruginosa*, biofilm, persistence, extracellular matrix, ciprofloxacin, stringent response

## Abstract

Biofilms are commonly recalcitrant to antibiotics, through incompletely elucidated mechanisms such as tolerance and persistence. We aimed at investigating how a *Pseudomonas aeruginosa* biofilm escapes ciprofloxacin treatment. *P. aeruginosa* PA14 *in vitro* mature biofilms were challenged with supra-MIC ciprofloxacin concentrations. Cell viability was quantified by fluorescein diacetate assay. Population dynamics were determined by counts of surviving culturable cells. Biofilms were analyzed using confocal laser scanning microscopy (CLSM), and the expression of genes involved in stringent response, toxin-antitoxin HigB/HigA, and type 3 secretion system (T3SS) was quantified by RT-qPCR in untreated and treated biofilms. Ciprofloxacin exposure resulted in an initial reduction of bacterial counts following a biphasic time-kill curve. After 24 h of treatment, the overall cell activity and the density of culturable cells significantly decreased as compared to untreated biofilm. No resistant mutant was isolated among the <1% surviving cells. Phenotypic adaptation toward persistence appeared to start after only 1 h of antibiotic exposure, by an overexpression of the genes involved in stringent response and in the toxin-antitoxin system, whereas the expression of genes encoding for the T3SS remained unchanged. After 4 h of ciprofloxacin exposure, stringent response genes returned to their basal level of expression. After a prolonged ciprofloxacin exposure, a deep alteration in the matrix structure that became thinner and lost mushroom-like aggregates was observed, in relation with reduced biovolumes of exopolysaccharides and extracellular DNA. These results support that ciprofloxacin might first induce the bacterial killing of most bacterial cells, but simultaneously activate stringent response mechanisms contributing to the switch of a subpopulation toward a persister phenotype. Once the persister phenotype is expressed, and despite an unexpected alteration of the biofilm matrix, ciprofloxacin fails to eradicate biofilm.

## Introduction

*Pseudomonas aeruginosa* infections associated with indwelling medical devices are particularly difficult to treat because of *P. aeruginosa* ability to produce biofilm protecting from host defenses and chemotherapy. Thus, treating such infections without mechanical biofilm dispersion remains challenging (Mulcahy et al., 2014; Høiby et al., 2015). Among the antibiotics required to treat *P. aeruginosa* biofilm infections, ciprofloxacin is widely used (Mensa et al., 2018; Masadeh et al., 2019), being the sole oral anti-*P. aeruginosa* antibiotic, and diffusing in the biofilm deepest layers (Anderl et al., 2000; Walters et al., 2003). Despite these properties, previous studies demonstrated that ciprofloxacin failed to eradicate *P. aeruginosa* biofilms, even in experimental conditions where no resistant mutants were selected (Spoering and Lewis, 2001; Pamp et al., 2008; Benthall et al., 2015; Pawar et al., 2015). Indeed, the treatment failure frequently resulted from the selection of persister cells. Such persisters derive from fully susceptible strains (Balaban, 2004) that become recalcitrant to antibiotics after a phenotypic and reversible switch, induced by environmental factors, starvation, and several other active or passive mechanisms (Lewis, 2010; Lebeaux et al., 2014; Ciofu and Tolker-Nielsen, 2019). Though not fully elucidated, it is suggested that persistence may result from a reduced metabolic activity of cells in biofilm after the induction of stringent response, SOS response, toxin-antitoxin modules or even other unknown mechanisms (Mah and O’Toole, 2001; Viducic et al., 2006; Amato et al., 2014; Harms et al., 2016).

Because the mechanisms and kinetics of *P. aeruginosa* biofilm tolerance to ciprofloxacin are still incompletely unraveled, we investigated the impact of ciprofloxacin on biofilm structure and the switch phenomenon toward persistence of biofilm-embedded cells by confocal laser scanning microscopy (CLSM) and transcriptomic analysis.

## Materials and Methods

### Bacterial Strain and Biofilm Model

The experiments were performed with the wild-type PA14 reference strain. The ciprofloxacin MIC and mutant prevention concentration for PA14 were, respectively 0.125 and 4 mg/L, determined as previously described (Wiegand et al., 2008; Díez-Aguilar et al., 2015). The mutant prevention concentration is the concentration that inhibits growth of the least, first-step mutant, corresponding experimentally to the lowest concentration that allows no colony growth when more than 10^10^ cells are applied to drug [ ciprofloxacin ]-containing agar plates (Drlica, 2003). For the biofilm model, overnight cultures of *P. aeruginosa* PA14 strain in Mueller-Hinton broth (MHB) were diluted to an optical density of 600 nm of 0.01 in MHB. Three mL of the bacterial suspension were inoculated in 6-well polystyrene culture plates and incubated under static conditions at 37°C for 1 h. Then, the suspension was removed and replaced by 3 mL of MHB. After 48 h of biofilm formation, wells were washed twice with 1 mL 0.9% NaCl to remove planktonic cells, and challenged with ciprofloxacin (Sigma-Aldrich, France) during 24 h.

### Evaluation of Cell Viability in Biofilms by Fluorescein Diacetate Assay

The fluorescein diacetate (FDA) assay relies on the cleavage of non-colored FDA by esterases of metabolically active viable bacteria into yellow fluorescein. FDA was added to treated biofilm after 24 h of ciprofloxacin exposure to measure the overall biofilm cell activity and compared with untreated biofilm. FDA (Sigma-Aldrich, France) was dissolved in acetone at a concentration of 1 mg/mL. A 1:5 FDA (v/v) working solution in 0.9% NaCl was freshly prepared before each assay. Biofilms were formed as previously described. Then, 2-day-old biofilms unexposed and exposed to ciprofloxacin (4–256 mg/L) during 24 h were rinsed with 1.5 mL of 0.9% NaCl, before addition of 1.5 mL FDA working solution. Plates were incubated in the dark at 37°C and absorbance was measured at 490 nm after 240 min. Control consisting of MHB without bacterial suspension was performed. Data were obtained from three independent biological replicates (Chand et al., 1994; Wanandy et al., 2005; Peeters et al., 2008).

### Count of Surviving Culturable Cells in Biofilm, After Ciprofloxacin Treatment in a Biofilm Time-Kill Assay

*Pseudomonas aeruginosa* PA14 biofilms were prepared as described above for colony counts. The 2-day-old biofilms unexposed and exposed to ciprofloxacin (4 mg/L) during 24 h were then dispersed mechanically by vortex and gentle sonication. Cells were numbered at 1, 2, 4, 8, 12, and 24 h of ciprofloxacin exposure onto MH2 plates without antibiotic or with ciprofloxacin at 0.5 mg/L to detect fourfold MIC resistant mutants. The ciprofloxacin MICs for surviving sessile cells recovered from MH2 plates after 24 h of ciprofloxacin exposure were determined by Etest according to the manufacturer’s recommendations.

### Biofilm Analysis by Confocal Laser Scanning Microscopy

Two-day-old *P. aeruginosa* PA14 biofilms were prepared as described above on 6-well glass-bottomed microplates (NEST, Grosseron, France), exposed or not to 4 mg/L of ciprofloxacin for further 24 h and analyzed by CLSM. After ciprofloxacin treatment, biofilms were washed twice with 1 mL 0.9% NaCl. The remaining surface-attached biofilm biomass was then stained by adding (i) 50 nM of Syto-9 (Invitrogen^TM^ Molecular Probes^TM^), a green fluorescent dye for total cells, and 1 μg/mL of white calcofluor (Sigma-Aldrich), a blue florescent probe labeling cellulose and other exopolysaccharides with β-1,4 linkages, (ii) 50 nM of Syto-9 and 1 μM of 1,3-dichloro-7-hydroxy-9,9-dimethyl-2(9H)-acridinone (DDAO) (Invitrogen^TM^ Molecular Probes^TM^), a red fluorescent probe labeling extracellular DNA (eDNA) and (iii) the Live/Dead BacLight Bacterial Viability Kit^®^ for microscopy (Thermo Fisher Scientific) differentiating viable and dead bacteria. Two controls were performed to exclude ciprofloxacin fluorescence. First, the fluorescence of ciprofloxacin alone was evaluated at 405 and 488 nm and no fluorescence was observed, showing that there was no auto-fluorescence at these wavelengths for the ciprofloxacin. Then, the fluorescence of a known concentration of cellulose and white-calcofluor was evaluated in presence or not of ciprofloxacin: no difference was observed between the two conditions (data not shown), pointing that there was no interference of ciprofloxacin in CLSM assays. A 2-day-old unexposed biofilm was also compared to the 3-day-old unexposed biofilm for exopolysaccharides biomass. For visualization and processing of 3D images, the Zen 2.1 SP1 zen software^1^ (Carl Zeiss Microscopy) was used. The biomass (μm^3^) of the biofilms were measured using the COMSTAT2 software^2^ (Givskov et al., 2000).

Data were obtained from at least three independent biological replicates.

### RNA Extraction From Biofilm for Transcriptomic Analysis

*Pseudomonas aeruginosa* PA14 biofilms were prepared for RNA isolation as described above. After 1 and 4 h of ciprofloxacin exposure, the biofilm was removed from wells by gentle sonication during 90 s and scrapping with a sterile pipette tip. The scraped biofilm was centrifuged for 5 min at 8000 *g*. Cells from six wells of a 6-well culture plate were pooled. Finally, the pellet was resuspended in 1 mL 0.9% NaCl for two washing steps. For the unexposed biofilm, only one of the 6-well plate was used according the same protocol previously described. Total RNAs were extracted using the Nucleospin RNA kit (Macherey Nagel, Hoerdt, France) and further treated with DNase (TURBO DNase free Ambion, Thermo Fisher Scientific) according to the manufacturer’s instructions. RNA was quantified and analyzed for its quality using a BioDrop μLITE (BioDrop Ltd., Cambridge, United Kingdom), and absence of contaminating DNA was checked by qPCR using RsmZ RNAs.

### Quantification of Gene Expression in Biofilms by Reverse Transcription-Quantitative PCR

The levels of expression of nine genes listed in the result section were analyzed in 2-day-old biofilms exposed to ciprofloxacin (at 4 mg/L) for 1 or 4 h and compared to untreated biofilms. First-strand cDNA synthesis was performed with OMNISCRIPT reverse transcription kit (Qiagen^®^) using standard laboratory protocols. Purified RNAs were used for one-step reverse transcription (RT) and real-time PCR amplification. Primers are listed in Supplementary Table S1. The PCR cycling conditions were as follows: 95°C for 5 min and 40 cycles of 15 s at 95°C, 30 s at 55°C. A melt curve was run at the end to evaluate primer dimers and other artifacts. Relative quantification was carried out from three independent biological replicates. Data were normalized to 16S gene expression and fold changes were calculated according to the 2^–ΔΔ*Ct*^ method (Guyard-Nicodème et al., 2008). A gene was considered as overexpressed when the fold change was at least two-times higher in treated biofilms, than in untreated biofilms. The stability of the 16S gene under different conditions was confirmed by comparing the respective cycle thresholds (CTs): 22.00 ± 0.53 for untreated biofilm and 22.48 ± 0.96 for ciprofloxacin-treated biofilm (*p* > 0.05).

### Statistical Analysis

Results were analyzed using the unpaired Student’s *t* test. Statistical significance was accepted when *p* values were < 0.05.

## Results

### Cell Viability in Biofilm

Ciprofloxacin at supra-MIC concentrations reduced the cell viability of biofilm-embedded cells as demonstrated by the FDA assay. Without ciprofloxacin, the absorbance measured in untreated biofilm was 6.24 ± 1.28. After a 24 h ciprofloxacin exposure, at concentrations ≥ 4 mg/L, the signal was significantly reduced (*p* < 0.05). The absorbances were 0.74 ± 0.37, 0.31 ± 0.10, 0.09 ± 0.07, and 0.11 ± 0.07, respectively for ciprofloxacin concentrations of 4, 8, 96, and 256 mg/L (Supplementary Figure S1). The ciprofloxacin concentration of 4 mg/L, corresponding to the mutant prevention concentration, the through concentration in adults treated orally with 500 mg twice daily, and achieving in human the pharmacokinetic/pharmacodynamic (PK/PD) objectives associated with clinical efficacy for ciprofloxacin (AUC_0__–__24_/MIC ≥ 125), resulted in inhibition of cell multiplication, as attested by a low absorbance signal in the FDA assay.

Regarding the biofilm time-kill assay, starting from a 7.9 log_10_ cfu/mL mean inoculum, a 3.0 log_10_ cfu/mL bacterial reduction was achieved as soon after 1 h, followed by a 2.0 log_10_ cfu/mL progressive reduction from 1 to 12 h and lastly a 3.0 log_10_ cfu/mL plateau until 24 h (details in Supplementary Figure S2). Of note, no resistant mutant was detected among surviving bacteria (neither on plates supplemented with fourfold ciprofloxacin MIC nor by measurement of ciprofloxacin MIC for surviving sessile cells). Finally, after 24 h of ciprofloxacin treatment, both the absorbance signal and the inoculum of surviving culturable cells were significantly reduced, compared to untreated biofilms.

### Biofilm Architecture

**FIGURE 1:**
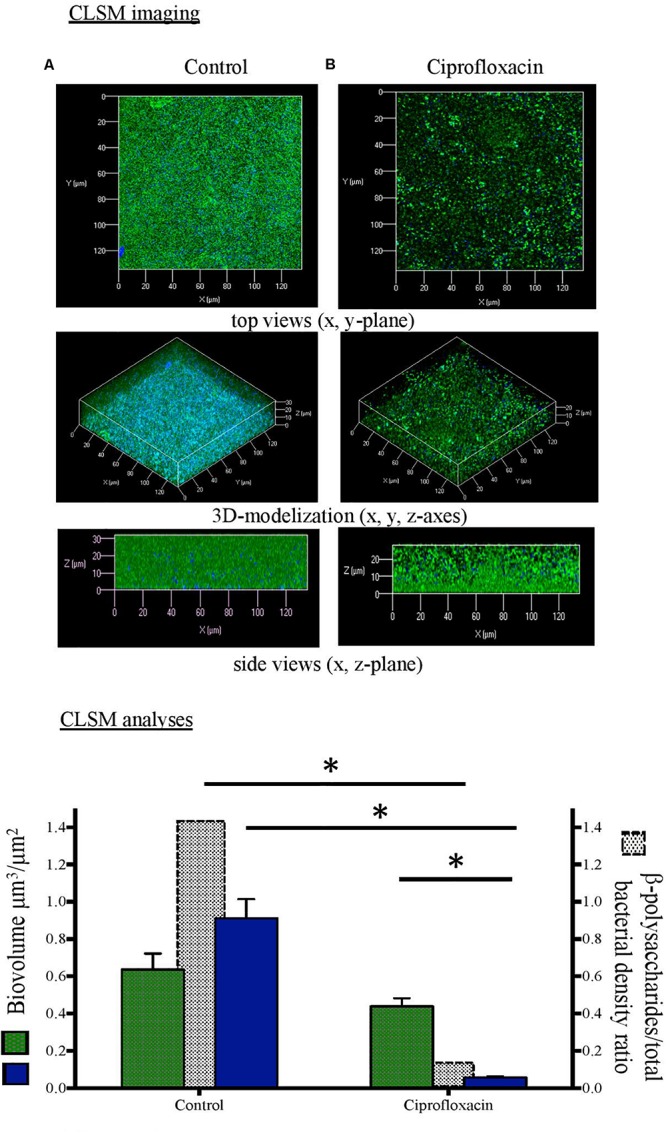
Confocal laser scanning microscopy (CLSM) analysis of *Pseudomonas aeruginosa* cells and β-polysaccharides in biofilm unexposed **(A)** or exposed to ciprofloxacin during 24 h **(B)**. CLSM imaging: total cells in green and β-polysaccharides in blue after staining with Syto-9 and white calcofluor. CLSM analyses: 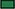 total cells (left *x*-axis), 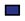
β-polysaccharides (left *x*-axis), 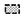
β-polysaccharides/total bacterial density ratio (right *x*-axis). ^∗^*p* < 0.05.

**FIGURE 2:**
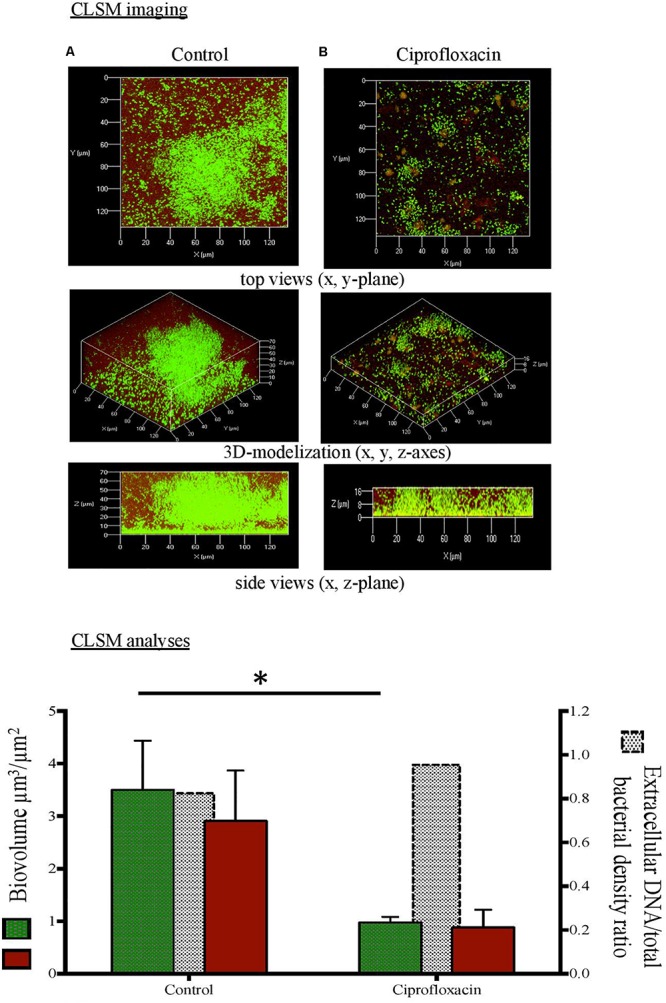
Confocal laser scanning microscopy (CLSM) analysis of Pseudomonas aeruginosa cells and extracellular DNA in biofilm unexposed **(A)** or exposed to ciprofloxacin during 24 h **(B)**. CLSM imaging: total cells in green and extracellular DNA in red after staining with Syto-9 and DDAO (1,3-dichloro-7-hydroxy-9,9- dimethyl-2(9H)-acridinone). CLSM analyses: 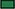 total cells (left *x*-axis), 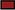 extracellular DNA (left *x*-axis), 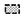 extracellular DNA/total bacterial density ratio (right *x*-axis). ^∗^*p* < 0.05.

**FIGURE 3:**
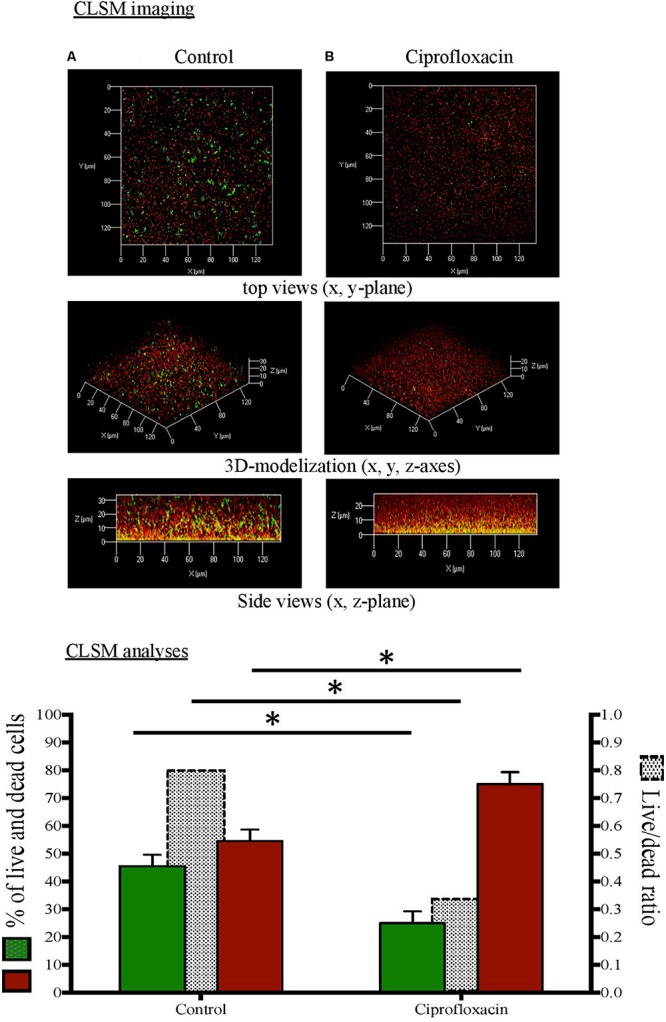
Confocal laser scanning microscopy (CLSM) analysis of live cells and dead cells in *Pseudomonas aeruginosa* biofilm unexposed **(A)** or exposed to ciprofloxacin during 24 h **(B)**. CLSM imaging: live cells in green and dead cells in red after staining with the Live/Dead BacLight Bacterial Viability Kit^®^. CLSM analyses: 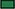 live cells (left *x*-axis), 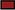 dead cells (left *x*-axis), 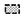 live/dead ratio (right *x*-axis). ^∗^*p* < 0.05.

As ciprofloxacin had a deleterious effect on cell viability, we therefore investigated the effect of the antibiotic exposure on biofilm architecture. Without ciprofloxacin, the 3-day-old biofilm was largely spread on the glass surface, was robust and compact with maximum and average thicknesses of 50.2 and 33.2 μm, respectively and mushroom-like aggregates of materials (Figures 1A, 2A, 3A). The biovolumes of β-polysaccharides and of total cells were stable between untreated 2- and 3-day-old biofilms (*p* > 0.05) (data not shown).

As compared to a 3-day-old untreated biofilm, ciprofloxacin exposure led to a sparser biofilm with a deep reduction in the biofilm thicknesses (maximum and average of 21.0 and 18.5 μm, respectively) and a disruption of the mushroom-likes structures (Figures 1B, 2B, 3B). Since the biofilm architecture seemed to be affected after ciprofloxacin exposure, the two major matrix components, i.e., exopolysaccharides (Figure 1) and eDNA (Figure 2), were explored as well as the ratio of live cells and dead cells (Figure 3) by specific staining. As shown Figure 1, the blue fluorescence was significantly less visible after ciprofloxacin exposure suggesting alterations in the exopolysaccharides of the matrix. COMSTAT analyses revealed that the biovolume of β-polysaccharides decreased markedly from 0.91 ± 0.10 μm^3^ in the untreated biofilm to 0.06 ± 0.01 μm^3^ (*p* < 0.05) and the ratio exopolysaccharides/total bacterial density declined from 1.43 ± 0.04 to 0.13 ± 0.03 (*p* < 0.05). As shown Figure 2, the red fluorescence labeling eDNA was less intense after ciprofloxacin exposure and holes in the biofilm structure were observed. COMSTAT analyses showed that the biovolume of eDNA decreased from 2.91 ± 0.96 μm^3^ to 0.88 ± 0.34 μm^3^ (*p* = 0.05) whereas the ratio eDNA/total bacterial density remained stable (0.84 ± 1.12 versus 0.96 ± 0.14, *p* = 0.7). The analysis of cell viability using the Live/Dead staining kit (Figure 3), showed that the red fluorescence corresponding to dead cells was more intense in biofilms exposed to ciprofloxacin than in controls. Indeed, COMSTAT analyses quantified a 1.8-fold decrease in the percentage of live cells after ciprofloxacin exposure (live cells decline from 45.5 ± 4.1 to 25.0 ± 4.3%, *p* < 0.05) and a 1.4-fold increase in the percentage of dead cells, indicating the ciprofloxacin deleterious effect, in accordance with our previous results obtained with FDA assay and cell counts. However, though ciprofloxacin resulted in major bacterial killing and structural disruption of the biofilm matrix, susceptible cells still survived in biofilm after antibiotic exposure.

### Gene Expression in Biofilm

To gain further insights into the adaptive mechanisms underlying *P. aeruginosa* persistence in biofilm upon ciprofloxacin exposure, we measured the dynamics in the expression of genes potentially involved in antibiotic tolerance (i) three stringent response genes (*spoT*, *relA*, and *lon*) known to be involved in biofilm tolerance to antibiotics, (ii) the two genes of the HigB-HigA toxin-antitoxin system (*higB* and *higA*) potentially involved in persister formation and the four genes involved in the type III secretion system (T3SS, *exsA*, *exsC*, *exoU*, and *pcrV*) a key virulence factor of *P. aeruginosa*. All these genes were quantified directly from the biofilm, before antibiotic exposure, and after 1 and 4 h of ciprofloxacin exposure at 4 mg/L.

**FIGURE 4:**
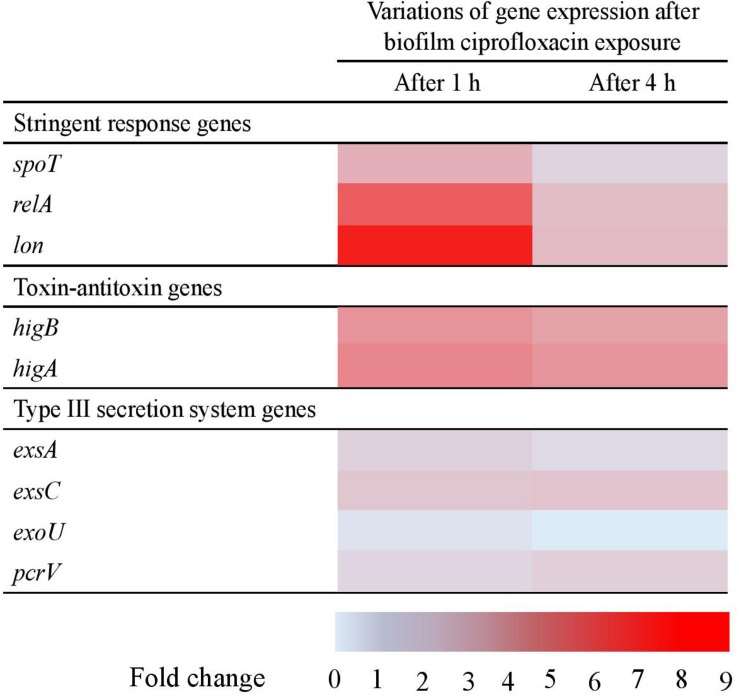
Levels of expression of stringent response, toxin-antitoxin and type III secretion system genes in *Pseudomonas aeruginosa* biofilm after 1 and 4 h of ciprofloxacin exposure, compared with unexposed biofilm. Results represent the means from three independent experiments. No statistical differences (*p* > 0.1).

As presented in a “heat map” that portrayed the trends in the variations of gene expressions obtained from the three biological replicates (*p* > 0.1) (Figure 4) and as compared to untreated biofilms, ciprofloxacin seemed to change the gene expression in biofilm after 1 h of exposure. A moderate increase in the expression of the three stringent response genes was measured, respectively a mean 2. 8-, 6. 4-, and 9.1-fold increase for *spoT*, *relA*, and *lon*. The two toxin-antitoxin system genes *higB* and *higA* were both slightly more expressed with respectively a 4.1- and 4.6-fold increase, whereas the level of expression of T3SS-related genes remained stable.

After 4 h of ciprofloxacin exposure, the T3SS genes were still not affected and the toxin-antitoxin genes expression remained overexpressed at the same level (∼fourfold), whereas the genes involved in the stringent response were no longer significantly up-regulated (from 1.4 to 2.4-fold). Thus, the dynamic of genes expression revealed a very rapid adaption after ciprofloxacin exposure as stringent response related-genes being overexpressed as early as 1 h of antibiotic.

## Discussion

Whilst it is now known that biofilm infections are difficult to eradicate because of their enhanced tolerance to antimicrobials (Costerton et al., 1999; Donlan, 2001), the switch toward a persistent phenotype is still incompletely elucidated. In our work, and as expected, CLSM analyses performed before any antibiotic exposure, portrayed a biofilm composed of bacterial cells entrapped in a cohesive and structurally robust matrix made of eDNA and exopolysaccharides (Limoli et al., 2015; Moradali et al., 2017). Consistent with other works, ciprofloxacin induced a rapid decrease in the bacterial density, followed by a survival of a subpopulation not eradicated despite prolonged antibiotic exposure (Pamp et al., 2008; Mulcahy et al., 2010; Reffuveille et al., 2014; Pawar et al., 2015). Noticeably, a 24 h ciprofloxacin exposure induced a deep disruption of the biofilm matrix. It was recently described that the β-lactam ceftazidime may weaken the polysaccharide matrix synthesis of *P. aeruginosa* PAO1 through a reduction in the production of Pel and Psl exopolysaccharides (Otani et al., 2018). Yasuda et al. (1993) also showed that clarithromycin treatment reduced the quantity of alginate and hexose and resulted in eradication of the membranes structures of the biofilm. Pel, the unique exopolysaccharide produced by *P. aeruginosa* PA14 (Colvin et al., 2011), is known to cross-link eDNA in the biofilm and is instrumental in its ability to interact with other key biofilm matrix components. Thus, reducing Pel amount might lead to disrupt the biofilm matrix. In our study, the biomass of exopolysaccharides remained stable between day 2 and day 3 in untreated biofilms, but was strongly reduced at day 3 after 24 h of ciprofloxacin exposure. Such a reduced biomass is probably partly related to the reduction of the bacterial density. But the high reduction of the ratio exopolysaccharides/total bacterial density after 24 h of ciprofloxacin exposure suggests that the production of exopolysaccharides by surviving cells might also be impaired. The mechanism underlying such reduction of exopolysaccharide production after ciprofloxacin exposure remains unknown, and investigating the regulatory cascade leading to Pel production would surely be promising research track.

In our study, the time-kill assay showed a substantial reduction of the viable cells after ciprofloxacin treatment and the analysis by CLSM using the Live/Dead BacLight Bacterial Viability Kit^®^ indicated an increase of dead cells after 24 h of ciprofloxacin exposure. Even if ciprofloxacin does not directly induce lysis of bacterial cells as do beta-lactams by interfering in cell wall biosynthesis, it has been shown that cell death that is induced by fluoroquinolones and especially that ciprofloxacin can be associated with formation of vacuoles and cell lysis resulting in extrusion of intracellular contents (Elliott et al., 1987). In our biofilm model, we might so have expected a release of DNA after the death of biofilm cells induced by ciprofloxacin exposure. But a decrease in the biovolume of eDNA was here observed, together with a reduction in the biovolume of exopolysaccharides as reported above. Since Pel colocalizes and physically interacts with eDNA in biofilm (Jennings et al., 2015), it can be suggested as an hypothesis that the eDNA could not be retained and entrapped in the biofilm because of the deep decline in the β-polysaccharides.

Despite such a disruption of the biofilm structure, ciprofloxacin failed to eradicate biofilm as evidenced by colony counts and live/dead staining after 24 h of ciprofloxacin exposure. The fluorescence assay, used to evaluate cell viability (Peeters et al., 2008), revealed an intense cell activity in unexposed biofilm, markedly reduced after ciprofloxacin exposure. Accordingly, antibiotic-induced dormancy has been previously shown as presumably one of the mechanisms involved in the antibiotic tolerance of biofilms (Amato et al., 2014). To further investigate the molecular mechanisms involved in the switch toward such a reduced metabolic activity after antibiotic exposure, the expression of stringent response genes was measured directly from biofilm. As soon as 1 h after the onset of ciprofloxacin exposure, stringent response genes *spoT*, *relA* and the gene encoding the protease Lon were largely overexpressed, while expression of virulence genes such as *exsA*, *exsC*, *exoU*, and *pcrV* encoding for the T3SS was largely unchanged. Stringent response can induce reduced metabolic activity particularly in biofilm and hence contribute to biofilm tolerance. Indeed, previous works have demonstrated that a deficient *P. aeruginosa* double mutant *ΔrelAΔspoT* impaired in its ability to produce the stringent response signaling compound (p)ppGpp demonstrated a lower tolerance to ofloxacin (Nguyen et al., 2011; Stewart et al., 2015). Conversely, the *P. aeruginosa* T3SS has been shown to essentially act as a pathogenetic and virulence factor (Diaz and Hauser, 2010). Recently, Li et al. (2016) demonstrated that sub-inhibitory ciprofloxacin concentrations displayed increased cytotoxicity, depending on the up-regulation of the T3SS in planktonic cultures. In our biofilm experiment, and after exposure to higher and effective ciprofloxacin concentrations, T3SS was not up-regulated. Hence, T3SS didn’t seem to play a role in antibiotic tolerance in biofilm. In line with these results, it can be suggested that sub-inhibitory antibiotic concentrations might trigger defense mechanisms in which virulence pathways such as T3SS might be involved, whereas presumably lethal antibiotic concentrations would lead to a general stress response activation in which starvation strategies such as stringent response might be “the last hope” for bacterial survival (Fajardo and Martínez, 2008; Andersson and Hughes, 2014; Harms et al., 2016).

Regarding the toxin-antitoxin systems, their role in *P. aeruginosa* biofilm persistence still remains elusive (Maisonneuve et al., 2018). Recently, Guo et al. (2019) demonstrated that HigA, when produced at a higher level than HigB, repressed virulence gene expression such as *mvfR* which controls the synthesis of pyocyanin. Moreover, in the presence of gentamycin or ciprofloxacin, the Lon protease was activated, leading to the degradation of HigA and the derepression of *higB* transcription. During the first stages of ciprofloxacin exposure in our biofilm model, *higA* and *higB* genes were overexpressed all along the experiment and each other at a comparable level. This result suggests that the antitoxin protein might inhibit her cognate toxin and act as a transcriptional repressor to control the production of virulence factors. Our results differed from those of reported by Li et al. (2016) and Guo et al. (2019) since *higA* was upregulated in our work despite a *lon* overexpression. This difference in the transcriptomic profiles of toxin-antitoxin system might depend on the experimental conditions retained in the studies. Our transcriptomic analysis was performed directly from biofilm after exposure to supra-MIC ciprofloxacin concentrations whereas the studies reported above were achieved in planktonic cultures after exposure to sub-inhibitory antibiotic concentrations. We therefore assume that either the HigA degradation will occur after a longer antibiotic exposure in biofilm or that the toxin HigB does not contribute to biofilm persistence.

A hypothesis of this study was the change in the gene expression profile that happened very early after antibiotic exposure, particularly for the stringent response, and that could be involved in a phenotypic switch toward antibiotic recalcitrance and reduced cell activity. After the initiation of stringent response, the switch for persistence and then dormancy might be completed, and stringent response might no longer be essential, as indicated by the decrease of the stringent response genes expression after an extended ciprofloxacin exposure in our study. Thus, our data highlighted that the general stress response seemed to be triggered very rapidly after the antibiotic treatment. To our knowledge, no other study has reported the dynamic adaptation of *P. aeruginosa* over time in biofilm in the presence of ciprofloxacin. Nevertheless, further studies are needed to deepen this issue. For example, it could be very informative to assess deficient *P. aeruginosa* mutants in the genes *spoT*, *relA*, and *lon* upon ciprofloxacin treatment in our biofilm model and in other *in vitro* and *in vivo* biofilm models.

Although this study provides new findings regarding the impact of ciprofloxacin in antibiotic recalcitrance in biofilm, results should be interpreted with caution. First, this study was limited to the study of a single strain. Moreover, mimicking the biofilm-life style of bacteria remains an arduous task (Lebeaux et al., 2013). The results obtained in biofilm models might largely vary, depending on strain, antibiotic, medium used, and many other experimental conditions (Macia et al., 2014). Then, the *P. aeruginosa* metabolism depends on numerous, highly regulated pathways. Thus, a complete transcriptomic profile would have been more informative. Unfortunately, and like other authors (Jahns et al., 2016), despite repeated attempts we were not able to extract enough RNA to perform RNAseq analysis.

In summary, the current study demonstrated that ciprofloxacin may be responsible of initial bacterial killing of a large part of the biofilm bacterial population. But, concurrently, ciprofloxacin may induce in a fraction of the initial population an activation of stringent response starting as early as 1 h after antibiotic exposure. Stringent response, probably combined with other unelucidated regulatory mechanisms, seemed to contribute to a switch toward a persister, antibiotic recalcitrant, phenotype of surviving cells. After 24 h of antibiotic treatment, and despite an unexpected disruption of the biofilm matrix by ciprofloxacin, persister cells were not eradicated. Even if this study still does not provide a definitive mechanistic explanation for the biofilm-related tolerance to antibiotics, it might contribute to a better understanding of the bacterial phenotypic switch toward persistence during biofilm-related infections.

## Data Availability Statement

All datasets generated for this study are included in the article/Supplementary Material.

## Author Contributions

AS, ME, and SC designed the study. AS, VR, EB, and MB performed the experiments. AS, ME, EB, and SC analyzed the data. AS and ME wrote the manuscript. FC reviewed the manuscript and figures. All authors amended and approved the final version of the manuscript.

## Conflict of Interest

The authors declare that the research was conducted in the absence of any commercial or financial relationships that could be construed as a potential conflict of interest.

## Footnotes

1 https://www.zeiss.com/microscopy/int/downloads/zen.html

2 http://www.imageanalysis.dk/
