# Supplementary figures and images for: Understanding Ciprofloxacin Failure in Pseudomonas aeruginosa Biofilm: Persister Cells Survive Matrix Disruption
Source: Front Microbiol. 2019 Nov 13;10:2603. doi: 10.3389/fmicb.2019.02603 (PMC6864029; doi:10.3389/fmicb.2019.02603)

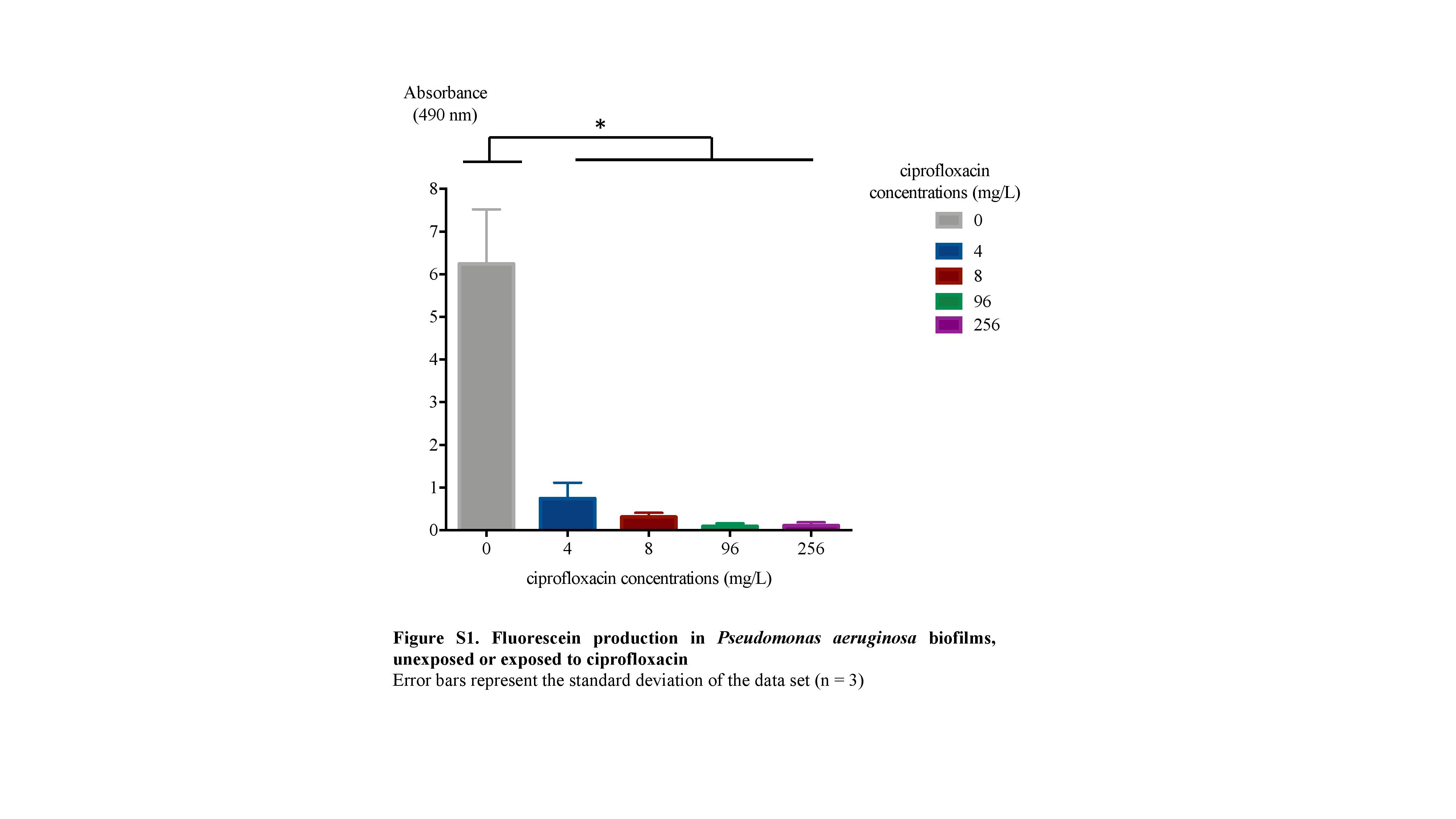

Supplement: Supplementary file 1 [file Image_1.JPEG]

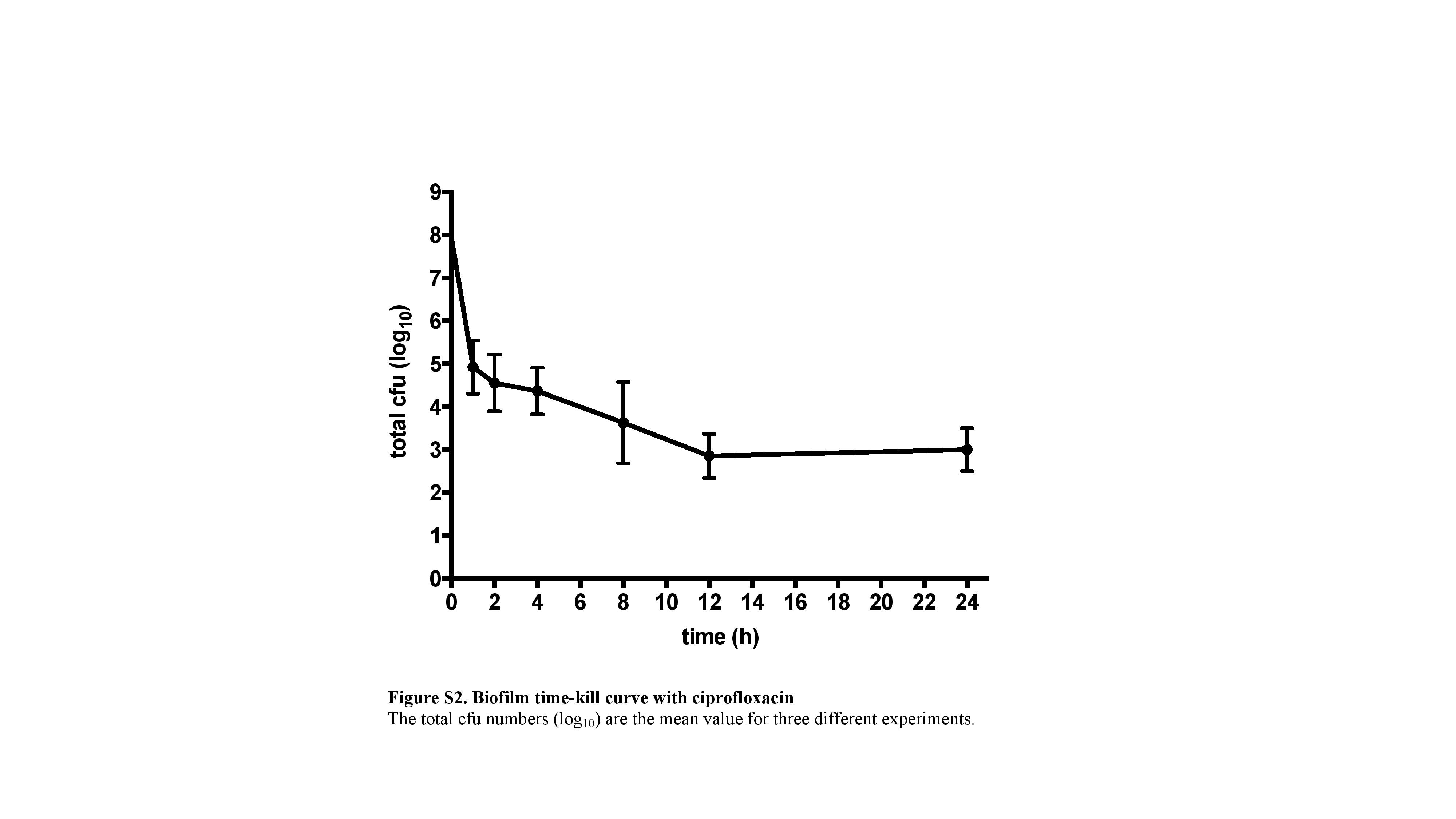

Supplement: Supplementary file 2 [file Image_2.JPEG]
