# Supplementary material for: Understanding Ciprofloxacin Failure in Pseudomonas aeruginosa Biofilm: Persister Cells Survive Matrix Disruption
Source: Front Microbiol. 2019 Nov 13;10:2603. doi: 10.3389/fmicb.2019.02603 (PMC6864029; doi:10.3389/fmicb.2019.02603)
Supplement: Supplementary file 3 [file Table_1.DOCX]

**Table S1. Primers used in the study for the quantification of the gene expression**

| **Function and Primer** | **Nucleotide sequence (5’-3’)** |
| --- | --- |
| 16S F | ATC TTC GGA CCT CAC GCT ATC |
| 16S R  exoU F  exoU R | TCA TCC TCT CAG ACC AGT TAC  GTT GAG TGC TTA CAT TCC  TTG AAC ACC ACT AAT TGC |
| exsA F | GCT ATG TCG TAA GTA CCA |
| exsA R | GAA GCC TTG TAG AAA CTG |
| exsC F | ATG GAT TTA ACG AGC AAG GTCA A |
| exsC R | GAG GGA CAG GGA AGG CAA A |
| higA F  higA R  higB F  higB R | GTT TCT GAT GGA GTT GGA T  ATA TCG TTC ACT GTC GGA  TTT GAG ACG GGT CTT TCG  TAG CCG CAT GAA GCA TTG |
| lonF | TAT CTC GCC GTG CAA AAG C |
| lonR  rsmZ F  rsmZ R | CCC CAC CAG GCA AAG GA  CGTACAGGGAACACGCAAC  ATTACCCCGCCCACTCTTC |
| pcrV F  pcrV R | CAC GCT CTA TGG CTA TGC  AAG GTA TCC AGA TTG CTC AG |
| relA F  relA R  spoT F | CGC GAT CCG TGC CTA TG  CAG AAC GTT GAT CCG CTC ATT  AAG GCG TTC AAC GAG ATC ATG |
| spot R | CCC AGT ACG CGAT AGC AGG TA |
